# Patisiran in ATTRv amyloidosis with polyneuropathy: “PatisiranItaly” multicenter observational study

**DOI:** 10.1007/s00415-025-12950-3

**Published:** 2025-02-15

**Authors:** Vincenzo Di Stefano, Pietro Guaraldi, Angela Romano, Giovanni Antonini, Alessandro Barilaro, Chiara Briani, Marco Burattini, Ilaria Cani, Giulia Carlini, Marco Ceccanti, Vittoria Cianci, Pietro Cortelli, Marco Currò Dossi, Daniela Di Lisi, Antonio Di Muzio, Yuri Falzone, Massimiliano Filosto, Sabrina Gasverde, Chiara Gemelli, Luca Gentile, Mariangela Goglia, Luca Leonardi, Simone Longhi, Antonio Lotti, Fiore Manganelli, Anna Mazzeo, Giammarco Milella, Giuseppina Novo, Silvia Fenu, Giovanni Palumbo, Cristina Petrelli, Loris Poli, Luca Guglielmo Pradotto, Massimo Russo, Alessandro Salvalaggio, Maria Ausilia Sciarrone, Luigi Sellitti, Matteo Tagliapietra, Stefano Tozza, Mara Turri, Lorenzo Verriello, Francesca Vitali, Filippo Brighina, Marco Luigetti

**Affiliations:** 1 Department of Biomedicine, Neuroscience and Advanced Diagnostics (BIND), University of Palermo, Palermo, Italy; 2 IRCCS Istituto delle Scienze Neurologiche di Bologna, Bologna, Italy; 3 Dipartimento di Neuroscienze, Organi di Senso e Torace, UOC Neurologia, Fondazione Policlinico Universitario Agostino Gemelli IRCCS, Largo Agostino Gemelli, 8, 00168 Rome, Italy; 4 Department of Neurology Mental Health and Sensory Organs (NESMOS), Faculty of Medicine and Psychology, ‘Sapienza’ University of Rome and UniCamillus-Saint Camillus International University of Health Sciences, Rome, Italy; 5 AOU Careggi and Department of Neurosciences, Drug and Child Health, University of Florence, Florence, Italy; 6 Neurology Unit, Department of Neuroscience, University of Padua, Padua, Italy; 7 Neurology Unit, Ospedale Santa Croce di Fano, Fano, Italy; 8 Neurological Clinic, Department of Experimental and Clinical Medicine, Marche Polytechnic University, Ancona, Italy; 9 Department of Human Neuroscience, Sapienza University of Rome, Rome, Italy; 10 Neurology Unit, Great Metropolitan Hospital “Bianchi Melacrino Morelli”, Reggio Calabria, Italy; 11 Department of Neurology, Infermi Hospital, Rimini, Italy; 12 Division of Cardiology, University Hospital Paolo Giaccone, Palermo, Italy; 13 Department of Neuroscience, Imaging and Clinical Sciences, “G. D’Annunzio” University, Chieti, Italy; 14 Division of Neuroscience, Department of Neurology, Institute of Experimental Neurology, San Raffaele Scientific Institute, Milan, Italy; 15 Department of Clinical and Experimental Sciences, University of Brescia, Brescia, Italy; 16 NeMO-Brescia Clinical Center for Neuromuscular Diseases, Brescia, Italy; 17 ASL TO4, Ciriè, Italy; 18 IRCCS Ospedale Policlinico San Martino, Genoa, Italy; 19 Department of Clinical and Experimental Medicine, University of Messina, Messina, Italy; 20 Neuromuscular Diseases Unit, Department of Systems Medicine, Tor Vergata University of Rome, Rome, Italy; 21 Neuromuscular and Rare Disease Centre, Neurology Unit, Sant’Andrea Hospital, Rome, Italy; 22 Cardiology Unit, Cardiac Thoracic and Vascular Department, IRCCS Azienda Ospedaliero-Universitaria di Bologna, Bologna, Italy; 23 Department of Neuroscience, Reproductive and Odontostomatological Science, University of Naples ‘Federico II’, Naples, Italy; 24 Neurology Unit, Department of Basic Medical Sciences, Neurosciences and Sense Organs, University of Bari Aldo Moro, Bari, Italy; 25 S.C. Malattie Neurologiche Rare, Dipartimento di Neuroscienze Cliniche, Fondazione IRCCS Istituto Neurologico Carlo Besta, Milan, Italy; 26 Neurology Unit, AV3, ASUR Marche, Macerata, Italy; 27 Unit of Neurology, ASST Spedali Civili, 25100 Brescia, Italy; 28 Department of Neuroscience “Rita Levi Montalcini”, University of Turin, Turin, Italy; 29 IRCCS Istituto Auxologico Italiano, Piancavallo (Vb), Milan, Italy; 30 Department of Neuroscience, Università Cattolica del Sacro Cuore, Rome, Italy; 31 Department of Neuroscience, Biomedicina e Movimento, Università di Verona, Verona, Italy; 32 Dipartimento di Neurologia/Stroke Unit, Ospedale di Bolzano, Bolzano, Italy; 33 Neurology Unit, Department of Neurosciences, University Hospital Santa Maria della Misericordia, Udine, Italy

**Keywords:** Hereditary transthyretin amyloidosis, ATTRv-PN, RNA interference, Patisiran, Multicenter study, Real-world data

## Abstract

**Background:**

Hereditary amyloid transthyretin amyloidosis with polyneuropathy (ATTRv-PN) is a rare, inherited, multisystemic, progressive adult-onset disease, affecting sensorimotor nerves, and various organs. It is caused by mutations in the *TTR* gene, leading to misfolded monomers that aggregate, forming amyloid fibrils. Patisiran is a small, double-stranded interfering RNA encapsulated in a lipid nanoparticle, designed to enter hepatocytes and selectively target TTR mRNA to reduce both variant TTR and wild-type TTR (wt). This study presents a multicenter, real-life experience of patisiran’s effectiveness and safety in ATTRv-PN.

**Methods:**

We enrolled genetically confirmed ATTRv-PN patients from 29 specialized Italian centers. All subjects underwent neurological assessments, including familial amyloid polyneuropathy (FAP) staging, the Neuropathy Impairment Score (NIS), quality-of-life assessment using the Norfolk Quality of Life-Diabetic Neuropathy (Norfolk QOL-DN) questionnaire, and the Compound Autonomic Dysfunction Test (CADT). Additional assessments included baseline and follow-up measures of serum NT-proBNP and interventricular septal thickness.

**Results:**

A total of 181 ATTRv patients (69% male) were enrolled. Neurological onset was reported in 60.2% of cases. At baseline, 83.4% of patients exhibited multisystemic involvement, while only 16.6% presented isolated polyneuropathy. For approximately 70% of patients, patisiran was the first treatment; the remainder transitioned from tafamidis or inotersen. Following treatment, most patients demonstrated stabilization of neuropathy progression, regardless of baseline disease severity or genotype. The treatment was well-tolerated, with 90% of patients reporting no adverse events.

**Conclusion:**

Patisiran can be considered a valid therapeutic option for the management of patients with ATTRv amyloidosis. Considering its mechanism of action, similar outcomes could also be expected with the wider utilization of newly approved gene silencers for ATTRv therapy, such as vutrisiran.

**Supplementary Information:**

The online version contains supplementary material available at 10.1007/s00415-025-12950-3.

## Introduction

Hereditary amyloid transthyretin amyloidosis with polyneuropathy (ATTRv-PN, “v” for variant) is a rare, inherited, multisystemic, progressive adult-onset disease that affects sensorimotor nerves and various organs [1]. ATTRv-PN results from mutations in the *TTR* gene, which cause the aggregation of misfolded monomers and the formation of amyloid fibrils [2]. Patients with ATTRv-PN experience a wide spectrum of symptoms, including neurological (peripheral sensorimotor neuropathy, autonomic neuropathy, carpal tunnel syndrome, and lumbar spinal stenosis), cardiac (cardiomyopathy and arrhythmias), gastrointestinal (nausea, vomiting, early satiety, diarrhea, severe constipation, alternating diarrhea constipation, and involuntary weight loss), and renal manifestations (renal failure and proteinuria), among others [2]. The disease primarily causes progressive axonal sensorimotor polyneuropathy, which can severely impair mobility, often leading to wheelchair dependence or even confinement to bed [1, 3]. Disease severity is classified according to the Coutinho staging system, based on walking ability [2].

Early and accurate diagnosis is crucial due to the disease’s rapid progression [4]. However, diagnosing ATTRv-PN is challenging and often delayed by several years from symptom onset, as clinical presentation can vary widely, even among family members. [5–7]. Recent advancements in understanding ATTRv pathophysiology have accelerated research, leading to the development of new, effective therapies that significantly improve survival and quality of life for affected patients [8–11]. A few years ago, the 5-year survival rate for ATTRv patients was approximately 30%; however, innovative therapies now offer the potential to reduce mortality and better manage disease-related disabilities [9]. The Italian group specializing in peripheral nervous system diseases has developed a registry to study the epidemiological characteristics of ATTRv and genotype–phenotype correlations, identifying a relatively high prevalence compared to other non-endemic countries [12, 13].

Patisiran, a small, double-stranded interfering RNA encapsulated in a lipid nanoparticle, specifically targets hepatocytes where it binds to TTR mRNA, reducing both variant TTR and wild-type TTR (wt) [14, 15]. This innovative mechanism significantly lowers plasma TTR levels, effectively halting the progression of polyneuropathy and substantially improving both neuropathy severity and quality of life, as demonstrated in phase 3 randomized controlled clinical trial [16, 17]. Based on these results, patisiran was approved in February 2020 by the “Agenzia Italiana del Farmaco” (AIFA) for treating ATTRv amyloidosis with polyneuropathy in patients at FAP 1 and FAP 2 stages. The drug is currently prescribed through Regional Reference Centers for ATTRv diagnosis and treatment in Italy.

Subsequent studies have confirmed the safety and efficacy of patisiran over more than 12 months of treatment, regardless of concurrent or prior stabilizer therapy [18, 19]. Recent evidence also suggests a protective effect of patisiran on cardiac function [20–22]. Additionally, emerging data indicate that patisiran’s effectiveness may occur more rapidly than demonstrated in the pivotal trial [23], with greater efficacy observed in patients with lower disease burden, particularly regarding gastrointestinal symptoms [20, 24, 25].

## Patients and methods

### Study design and population

The objective of the present study is to confirm the efficacy and safety of patisiran in ATTRv amyloidosis with polyneuropathy by verifying the Italian experience, in a non-endemic area. The study was approved by the Ethical Committee of Palermo on 26th June 2023 (V n.6/2023), and it was conducted in conformity with the Declaration of Helsinki principles. The study was registered on the RSO national AIFA platform (ID815 Cod. PatisiranItaly23).

Patients with ATTRv amyloidosis and polyneuropathy treated with patisiran since February 2020 (following the drug’s approval in Italy) were enrolled from 29 specialized centers across Italy. The inclusion criteria were as follows: informed consent, age ≥ 18 years, diagnosis of symptomatic ATTRv hereditary amyloidosis with length-dependent sensorimotor neuropathy with a known amyloidogenic variant, and absence of other causes of neuropathy (e.g., diabetes, inflammatory polyneuropathy, AL amyloidosis, chronic alcoholism, neuropathy due to anti-MAG antibodies, or vitamin B12 deficiency). Patients also needed to be actively receiving patisiran according to Italian regulations (or during day hospital, or home infusion), with no concurrent therapies for ATTRv.

Exclusion criteria included lack of informed consent, discontinuation of patisiran treatment before 9 months, prior treatment with patisiran before 2020 in a clinical trial or under compassionate use, concurrent treatment with other drugs or participation in pharmacological clinical trials, active hepatitis B or C, and liver or kidney failure. Patients on patisiran were classified as “naive” if patisiran was their initial therapy for ATTRv-PN, or as “switch” if they began patisiran following another therapy.

All subjects underwent neurologic evaluations, clinical scales assessment, and serum biomarker determinations at the start of treatment (T0) and at 9-month intervals thereafter, in line with AIFA requirements (T1, 9 months; T2, 18 months; T3, 27 months; and T4, 36 months). Data were collected through the most recent available follow-up. For each patient, the following information was collected and analyzed: genotype, age at onset of symptoms, type of onset (neurological, cardiac, mixed, other), presence of an isolated neuropathy or of a neuropathy associated with multisystem involvement (cardiac, gastrointestinal, etc.) at baseline, age at diagnosis, age at the time therapy was initiated, “time-to-treatment” (time elapsed from symptom onset to the first drug received), “time-to-patisiran” (time elapsed from symptom onset to initiation of patisiran treatment), medication history (naive patient or switch from another treatment).

### Clinical evaluations (T0, T1, T2, T3, T4)

Each visit included neurological evaluation, clinical tests, clinical scales, instrumental examinations, and assessment of serum cardiac biomarkers, along with safety data.

Clinical tests and scales included:

- Familial amyloid polyneuropathy (FAP) stage: A 3-stage scale evaluating the overall severity of symptomatic polyneuropathy. FAP 1 stage refers to a symptomatic but fully ambulatory patient, FAP 2 stage is defined by the need for walking aids, and FAP 3 stage denotes wheelchair dependence or being bedridden.
- Neuropathy impairment score (NIS, range: 0–130): A composite score assessing the severity of neuropathy across sensory, reflexes, and motor domains. Higher scores indicate greater impairment, while a score of 0 represents normal function.
- Norfolk quality of life-diabetic neuropathy (QoL-DN) questionnaire (range: − 4 to 136): A patient-reported outcome measure evaluating the impact of neuropathy on quality of life, with higher scores denoting poorer QoL-DN.
- Compound autonomic dysfunction test (CADT): A scale assessing autonomic involvement across various domains and associated symptoms (range: 0–20 in males, 0–16 in females). Low scores correspond to severe dysfunction, while high scores represent normal values.

Instrumental exams included a transthoracic echocardiogram to assess the thickness of the interventricular septum (IVS). Serum cardiac biomarkers included the N-terminal pro B-type natriuretic peptide (NT-proBNP).

### Safety

All subjects enrolled in the study were included in the tolerability analysis. Adverse events were coded using the latest version of the MedDRA dictionary and summarized by system organ class (SOC) and preferred term (PT).

### Statistical analysis

The sample was described in its socio-demographic and clinical features using descriptive statistics techniques.

The patients were divided into subgroups according to the severity of the neuropathy at the start of patisiran treatment (evaluated based on baseline FAP stage: FAP stage 1 vs FAP stage 2), and genotype (p.Val50Met vs p.Phe84Leu *vs* p.Glu109Gln *vs* p.Ile88Leu + p.Val142Ile).

For continuous data, the normal distribution was assessed using the Shapiro–Wilk test and graphical methods (normal Q–Q plots, boxplots). Given the nature of the data, non-parametric tests were applied.

Comparisons between two independent groups were performed using the Mann–Whitney *U* test. Differences between three or more independent groups were evaluated with the Kruskal–Wallis *H* test; post-hoc pairwise comparisons, if pertinent, were assessed using Dunn’s test.

A Friedman test (and post-hoc tests, when necessary) was used to evaluate within-group changes over time. Given the small sample size at T4, repeated measures analyses were conducted from T0 (baseline) to T3 (i.e., the 27 month-evaluation).

Pairwise comparisons (for both between- and within-group analyses) were adjusted using the Bonferroni correction for multiple comparisons; adjusted *p* values (adj. *p*) are reported for post-hoc tests.

Correlations between clinical variables were examined using Spearman's rank-order correlation coefficient.

Statistical analysis was performed using IBM^®^ SPSS^®^ Statistics version 25.0 (IBM Corp., Armonk, NY, USA). For all analyses, the significance level was set at *α* ≤ 0.05.

## Results

### Study population

One hundred eighty-one ATTRv patients (69% male; mean age at the start of patisiran: 65.9 ± 11.7 years) were recruited. Neurological onset was observed in 60.2% of cases. At baseline, most patients (83.4%) presented multisystemic involvement, while only 16.6% presented with isolated polyneuropathy. Notably, no patient switched from a neurological phenotype to a mixed phenotype during the study. No deaths were observed during the study. Table 1 provides the demographics and clinical characteristics of the included ATTRv-PN patients. The less common genotypes are detailed in Supplementary Table 1.

**Table 1:** Demographic and clinical characteristics of the ATTRv patient cohort

	ATTRv patients (*n* = 181)
Males	124 (68.5%)
Positive family history	49 (27.1%)
Late-onset (> 50 years) patients	152 (83.9%)
Age at onset (years)	*M* = 62.85 ± 12.94; Mdn = 65.00 [56.00–73.00]
Age at the start of patisiran (years)	*M* = 65.93 ± 11.69; Mdn = 69.00 [59.25–75.00]
ATTRv onset	
Neurological	109 (60.2%)
Cardiological	51 (28.2%)
Mixed	8 (4.4%)
Other	13 (7.2%)
FAP stage at the start of patisiran (baseline, or T0)	
FAP1	130 (71.8%)
FAP2	51 (28.2%)
Available follow-up	
9 months (T1)	165 (91.2%)
18 months (T2)	110 (60.8%)
27 months (T3)	65 (35.9%)
36 months (T4)	16 (8.8%)
Genotype	
p.Phe84Leu	54 (29.8%)
p.Ile88Leu	38 (21.0%)
p.Val50Met	37 (20.4%)
p.Glu109Gln	15 (8.3%)
p.Val142Ile	7 (3.9%)
Other TTR variants (see Supplementary Table 1)	30 (16.6%)
Baseline NIS	*M* = 39.83 ± 31.54; Mdn = 31.00 [15.00–53.00]
Baseline Norfolk QoL-DN questionnaire	*M* = 44.34 ± 26.73; Mdn = 44.00 [21.00–66.00]
Baseline CADT	
M (*n* = 68)	*M* = 15.99 ± 3.71; Mdn = 17.00 [13.00–19.00]
F (*n* = 27)	*M* = 13.89 ± 2.68; Mdn = 15.00 [12.00–16.00]
IVS at baseline (mm) (*n* = 138)	*M* = 14.57 ± 3.98; Mdn = 14.10 [11.00–17.00]
NT-proBNP at baseline (ng/l) (*n* = 130)	*M* = 1219.99 ± 1875.84; Mdn = 525.50 [132.00–1525.00]

Categorical variables are expressed as absolute counts and percentages (%). Quantitative variables are reported as mean (*M*) ± standard deviation and median (Mdn) [interquartile range].

FAP, Familial amyloid polyneuropathyl NIS, Neuropathy Impairment Score; Norfolk QoL-DN, Norfolk Quality of Life–Diabetic Neuropathy questionnaire; CADT, Compound autonomic dysfunction test; M, Males; F, Females; IVS, Interventricular septum; NT-proBNP, N-terminal pro-B-type natriuretic peptide

### Safety

Patisiran demonstrated a favorable safety profile, with approximately 90% of patients experiencing no significant side effects. Adverse events considered related to study treatment, as well as those leading to study discontinuation, are summarized in Table 2.

**Table 2:** Adverse events (AEs) reported in our cohort of 181 ATTRv-PN patients treated with patisiran

Adverse events (AEs)	Frequency
Back pain	6 (3.3%)
Infusion site reaction	3 (1.7%)
Renal impairment	3 (1.7%)
Headache	2 (1.1%)
Itching	1 (0.6%)
Fatigue	1 (0.6%)
Abdominal pain	1 (0.6%)

### Clinical, neurophysiological and clinical scales data

Table 3 summarizes study cohort data at various time points from the start of patisiran (T0) to the last follow-up (T4).

**Table 3:** Clinical and instrumental data in the whole cohort of ATTRv patients at different time points, from the initiation of patisiran (T0) to the last follow-up (T4)

	T0	T1	T2	T3	T4
NIS	39.83 ± 31.54; 31.00 [15–53]	41.11 ± 31.21; 33.25 [16–61.50]	41.52 ± 33.94; 30.00 [14–64]	46.74 ± 34.66; 41.00 [16–65.75]	57.79 ± 30.86; 63.50 [40–90]
Norfolk QOL-DN	44.34 ± 26.73; 44.00 [21–66]	42.75 ± 26.92; 42.00 [20–62]	42.45 ± 28.78; 39.50 [18–66]	44.71 ± 27.08; 43.00 [20–72]	54.24 ± 33.95; 54.00 [22–80]
CADT (*M*)	15.99 ± 3.71; 17.00 [13–19]	16.20 ± 3.18; 16.00 [14–19]	16.35 ± 2.83; 16.00 [14–19]	16.41 ± 2.63; 16.00 [14–19]	15.88 ± 2.23; 16.00 [14.50–17.50]
CADT (*F*)	13.89 ± 2.68; 15.00 [12–16]	13.78 ± 2.34; 14.00 [12–16]	13.94 ± 1.89; 14.00 [13–16]	14.08 ± 1.78; 14.00 [12.50–16]	13.40 ± 1.67; 13.00 [12–14]
IVS (mm)	14.57 ± 3.98; 14.1 [11–17]	14.43 ± 3.91; 15.00 [12–17.1]	14.62 ± 3.42; 15.00 [12–17]	14.35 ± 3.09; 13.50 [12–17]	14.40 ± 2.84; 13.50 [13–17]
NT-proBNP (ng/l)	1219.99 ± 1875.84; 525.50 [132–1525]	1962.81 ± 5011.70; 360.50 [110–1500]	1921.77 ± 4976.77; 509.85 [99.45–1528]	1160.37 ± 2405.12; 415.00 [156–1177]	983.42 ± 1357.98; 330.50 [194–1219]

Specifically, T1 refers to the 9 month-evaluation, T2 to the 18 month-evaluation, T3 to the 27 month-evaluation, and T4 to the 36 month-evaluation.

All variables are reported as mean ± standard deviation and median [interquartile range]

NIS, Neuropathy Impairment Score; Norfolk QoL-DN, Norfolk Quality of Life-Diabetic Neuropathy questionnaire; CADT, Compound autonomic dysfunction test; M, Males; F, Females; IVS, Interventricular septum; NT-proBNP, N-terminal pro B-type natriuretic peptide

### Treatment choice

Patisiran was the initial treatment for 124 patients (68.5%), while it was used as a switch from a previous different therapy in approximately 30% of cases. Specifically, patisiran was initiated as the second treatment in 51 patients (28.2%) and as the third therapy in six patients (3.3%). No patient received a combination of patisiran and a stabilizer.

The mean delay between disease onset and the first treatment (time-to-treatment) across the entire cohort was 2.19 ± 3.42 years (median 1, IQR 0–3). When excluding patients with a family history, the time-to-treatment increased to 3.01 ± 3.68 years (median 2.00, IQR 1–4). When stratifying patients based on treatment history, the mean time-to-treatment was 2.34 ± 3.74 years (median 1.00, IQR 0.25–3) in “naïve” subgroup vs 1.88 ± 2.59 years (median 1.00, IQR 0–3) in the “switch” subgroup. In the latter, the mean time-to-patisiran was 5.16 ± 3.88 years (median 4.00, IQR 2–7).

### FAP stage

FAP remained stable in most cases after starting patisiran, with changes in the FAP stage rarely observed during follow-up; only twelve patients experienced stage progression (Supplementary Fig. 1).

### NIS

A Friedman test was performed on a subgroup of patients with complete data from T0 to T3 (*n* = 55) to evaluate changes in NIS scores over time following the initiation of patisiran. The results indicated a statistically significant difference in NIS scores across the different time points after treatment began (*p* = 0.004). Post-hoc analysis revealed a statistically significant change in NIS scores from T1 to T3 (median: 35.0 vs 40.0; adj. *p* = 0.021), and from T2 to T3 (median: 37.0 vs 40.0; adj. *p* = 0.042)”. At T3 (27 months follow-up), we observed that 10 out of 55 (18.2%) patients improved (NIS < baseline − 2 points), 24 out of 55 (43.6%) patients remained stable (NIS = baseline ± 2 points), and 21 out of 55 (38.2%) patients worsened (NIS > baseline + 2 points) [26].

### Norfolk QoL-DN

A Friedman test indicated a statistically significant change over time in Norfolk QoL-DN scores following the initiation of patisiran (*p* = 0.042). However, post-hoc analysis did not confirm any significant changes between the different time points, suggesting stabilization in quality-of-life measures after treatment initiation.

### CADT

No significant differences in CADT scores were detected over time for either males (*p* = 0.901) or females (*p* = 0.468).

### Cardiac parameters

Similarly, no significant changes were observed in IVS (*p* = 0.267) or NT-proBNP levels (*p* = 0.394) following the initiation of patisiran.

### Correlations at the baseline evaluation (T0)

In the whole cohort, baseline NIS showed a strong, positive correlation with baseline Norfolk QoL-DN scores (*ρ* = 0.708, *p* < 0.001). Additionally, both scales exhibited a moderate negative correlation with CADT scores in males (NIS: *ρ* = − 0.413, p < 0.001; Norfolk QoL-DN: *ρ* = − 0.511, *p* < 0.001), but not in females (NIS: *ρ* = − 0.375, *p* = 0.054; Norfolk QoL-DN: *ρ* = − 0.279, *p* = 0.160).

For cardiac parameters, a strong positive correlation was found between baseline IVS and NT-proBNP levels (*ρ* = 0.652, *p* < 0.001), as expected. Moreover, baseline IVS showed a weak/negligible negative correlation with both NIS (*ρ* = − 0.204, *p* = 0.016) and Norfolk QoL-DN (*ρ* = − 0.171, *p* = 0.048) in the overall cohort; however, no correlation was found between IVS and CADT scores in either male or female subgroup. In contrast, NT-proBNP levels did not exhibit any significant correlations with the neurological scales.

### Subgroups analyses

#### Stratification based on disease severity at the start of patisiran

Considering disease severity at the start of patisiran (evaluated in terms of baseline FAP stage, i.e. FAP stage at T0), patients classified as FAP1 at baseline exhibited lower NIS values compared to FAP2 patients, not only at T0 (median: 22.00 [IQR 12–39.25] vs 69.00 [48–95], respectively; *p* < 0.001), as expected, but also at each subsequent time point (*p* < 0.001 at T1, T2, and T3; Supplementary Table 2). In terms of progression over time within each subgroup, the Friedman test suggested an overall statistically significant change over time in the FAP1 subgroup (*p* = 0.027); however, post-hoc tests did not confirm any significance (all adj. *p* > 0.05). On the other hand, NIS values in FAP2 patients did not change significantly over time (*p* = 0.206).

Similarly, Norfolk QoL-DN scores were lower in FAP1 patients compared to FAP2 patients at baseline and each follow-up assessment (*p* < 0.001 at T0, T1, T2, and T3; Supplementary Table 2). However, Norfolk QoL-DN scores did not show significant changes over time in either FAP1 (*p* = 0.279) or FAP2 patients (*p* = 0.054).

As concerning CADT, FAP1 male patients had significantly higher values than FAP2 males at baseline (*p* = 0.022) and T2 (*p* = 0.010), but no significant differences were found at T1 (*p* = 0.065) or T3 (*p* = 0.277). CADT scores did not show significant variation over time in either the baseline FAP1 (*p* = 0.456) or FAP2 male groups (*p* = 0.479).

Due to the low number of available data, particularly in the baseline FAP2 female subgroup, no analysis was conducted on CADT scores for female patients.

### Stratification based on genotype

Genotype-based analysis was performed on the following subgroups: p.Phe84Leu, p.Ile88Leu + p.Val142Ile, p.Val50Met, and p.Glu109Gln. The p.Ile88Leu and p.Val142Ile variants were pooled into a single subgroup considering that they both share a predominant cardiological phenotype.

At the baseline evaluation (T0), NIS values differed significantly among genotypes (*p* < 0.001; Supplementary Table 3). Specifically, patients in the p.Ile88Leu + p.Val142Ile subgroup had lower NIS values (median: 15.00 [IQR 10–30]) compared to both p.Val50Met (median: 46.00 [IQR 21.50–68.50]; adj. *p* < 0.001) and p.Phe84Leu patients (median: 48 [IQR 32.25–76.63]; adj. *p* < 0.001). No other pairwise comparisons were significant. These differences were consistent across the follow-up periods (T1: *p* < 0.001; T2: *p* < 0.001; T3: *p* = 0.010; Supplementary Table 3).

Analogously, Norfolk QoL-DN scores at T0 showed significant differences between genotypes (*p* < 0.001; Supplementary Table 3). Patients in the p.Ile88Leu + p.Val142Ile subgroup reported lower scores (median: 26 [IQR 12–46.50]) compared to p.Val50Met (median: 50.00 [IQR 31–67]; adj. *p* = 0.028) and p.Phe84Leu patients (median: 56 [IQR 40.5–76.5]; adj. *p* < 0.001). Similar differences were also confirmed at each subsequent follow-up (T1: *p* < 0.001; T2: *p* = 0.010; T3: *p* = 0.016; Supplementary Table 3).

Regarding CADT in male patients, a statistically significant difference was evident at T0 (*p* = 0.004), T1 (*p* = 0.006), and T2 (*p* = 0.025); Supplementary Table 3. At T0, significant pairwise comparisons were found between the p.Ile88Leu + p.Val142Ile subgroup and the p.Phe84Leu subgroup (median: 19.50 [IQR 16.75–20] vs median: 16 [IQR 12–18], respectively; adj. *p* = 0.002). Conversely, at later time points (T1 and T2), the “cardiac genotype” group was significantly different from both the p.Phe84Leu (adj. *p* = 0.036 at T1; *p* = 0.038 at T2) and p.Val50Met subgroups (adj. *p* = 0.007 at T1; adj. *p* = 0.042 at T2). Due to the low number of patients with available data, no analysis could be conducted at T3 in males, and no analysis could be performed for females at any time points.

At baseline (T0), genotypes showed statistically significant differences in IVS values (*p* < 0.001; Supplementary Table 3). Specifically, p.Phe84Leu patients had lower IVS values (median: 12.00, IQR 11.00–13.00) compared to p.Val50Met patients (median: 14.50 [IQR 11.75–18.25]; adj. *p* = 0.050), and, even more so, compared to patients with known “cardiac” *TTR* variants (p.Ile88Leu + p.Val142Ile subgroup; median: 17.00 [IQR 15.00–19.00]; adj. *p* < 0.001), as expected. This difference remained significant at the 9-month (T1: *p* < 0.001) and 18-month (T2: *p* < 0.001) evaluations. However, in both cases, only the pairwise comparison between p.Phe84Leu and the cardiac variant group was confirmed as significant (T1: adj. *p* < 0.001; T2: adj. *p* < 0.001). No analysis could be conducted for the 27-month evaluation (T3) due to the low number of patients per subgroup with available IVS data.

Analogously, baseline NT-proBNP levels significantly differed among genotypes (*p* < 0.001; Supplementary Table 3). Patients with “cardiac *TTR* variants” (p.Ile88Leu or p.Val142Ile) had significantly higher NT-proBNP levels (median: 1831.00 [IQR 521.50–3518.50]) than p.Phe84Leu patients (median: 163.50 ng/l [IQR 62.45–885.00], adj. *p* < 0.001), as expected. Similar conclusions were observed at both T1 (*p* < 0.001) and T2 (*p* = 0.003), but not a T3 (*p* = 0.057) (Supplementary Table 3).

In terms of progression over time within each genotype subgroup, no significant changes were observed in neurological (NIS, Norfolk questionnaire scores, CADT in males) or cardiac parameters (IVS, NT-proBNP levels) in any of the analyzed subgroups (all *p* > 0.005).

Detailed statistical analyses are reported in Supplementary Tables 2 and 3.

## Discussion

Patisiran is a small, double-stranded interfering RNA encapsulated in a lipid nanoparticle that effectively penetrates hepatocytes, where it selectively targets TTR mRNA, leading to a reduction in TTR production [14, 17, 21]. The phase 3 APOLLO study confirmed the efficacy and safety of patisiran in patients with ATTRv amyloidosis, demonstrating its ability to halt or even regress disease progression in some cases [16]. Recent studies have also shown a correlation between improved functional and biochemical outcomes with regression of amyloid burden, especially at the cardiac level [15, 20, 21]. Literature data indicate that patisiran is effective and safe in alleviating both neurological and cardiovascular symptoms of ATTRv amyloidosis, while also maintaining a good quality of life, regardless of whether patients are in FAP stage 1 or 2, or the specific mutation involved [17, 21]. Moreover, its efficacy and safety have been confirmed for long-term use [18, 26, 27].

However, due to the rarity of ATTRv amyloidosis, there is still limited data on real-life experience with patisiran. Additionally, ATTRv amyloidosis is a heterogeneous disease, characterized by significant genotypic and phenotypic variability, making data from multicenter studies invaluable for investigating the effects of RNA silencers on specific and rare genotypes.

To our knowledge, this study, which collects data from 181 ATTRv-PN patients treated with patisiran, represents the larger cohort in real-world evidence studies. Our findings confirm the efficacy and safety of patisiran in ATTRv amyloidosis in a wide cohort of late-onset ATTRv-PN, with a long follow-up period of 36 months. Patisiran was found to be safe, with adverse events reported in only a very small percentage of patients (10%). Neuropathy remained stable at 27 months, as assessed by the CADT and Norfolk scales. Regarding the NIS scale, a slight progression was observed during follow-up. This change can be attributed to a few outliers who experienced significant deterioration during the follow-up period. Furthermore, we observed stabilization or improvement in NIS values at 27 months in approximately 60% of patients. Collectively, these results confirm data from recent real-life experience [26] exploring long-term treatment with patisiran, which showed the effect of RNA silencers in halting or slowing the progression of polyneuropathy. Indeed, a deterioration of 14.3 points per year is expected for a population with a median NIS of 32, based on a multinational natural history study [28]. Of note, the substantial stabilization from baseline obtained in CADT and Norfolk QoL-DN scores, as well as a noticeable slowing of NIS progression, at 27 months after the start of treatment is a significant outcome, considering the heterogeneous cohort with mixed genotypes and phenotypes.

The effect of patisiran on cardiac parameters has been confirmed by the APOLLO-B study; however, cardiac parameters are not frequently reported, and recent real-world studies are limited [20–22, 26]. Besides, the present study confirms the effects of RNA silencers on cardiac function by exploring echocardiographic parameters and serum biomarkers.

Interestingly, when stratifying the population by baseline disease severity according to FAP stage, we found no significant progression of neuropathy in either subgroup (FAP 1 and FAP2). This finding, apparently discordant with the overall population data, may indicate that the general results were influenced by a few outliers, further emphasizing the efficacy of patisiran regardless of baseline disease severity [26]. However, we cannot exclude that the lack of progression in individual subgroups could derive from the reduction in sample sizes increasing the risk of type 2 error.

Moreover, we did not observe any difference in disease progression after treatment when stratifying our population by genotype, suggesting that patisiran has the same efficacy in all analyzed *TTR* variants. Notably, we did not observe any progression of cardiac dysfunction parameters, either in patients with “cardiological” mutations or in those with other variants, confirming the effect of patisiran on cardiomyopathy [21]. Furthermore, we confirmed a possible neurological involvement in patients with “classically” cardiac mutations [29, 30].

Although our study has many strengths, we must acknowledge some limitations due to its retrospective design. These include the lack of important parameters such as TTR knockdown, PND stage, and the Composite Autonomic Symptom Score-31, which are fundamental tools for longitudinal patient monitoring.

## Conclusions

In conclusion, patisiran can be considered a valid therapeutic option for the management of patients with ATTRv amyloidosis. Our data demonstrate that patisiran is both safe and effective, generally resulting in stabilization of neuropathy progression and quality of life, regardless of baseline disease severity or genotype.

Given its mechanism of action, similar results could be expected also when the recently approved gene silencer, vutrisiran, becomes widely utilized.

## Supplementary Information

Below is the link to the electronic supplementary material.

Supplementary file1 (DOCX 51 KB)

Supplementary file2 (DOCX 17 KB)

Supplementary file3 (DOCX 26 KB)

Supplementary file4 (DOCX 29 KB)

## Data Availability

The data that support the findings of this study are available from the corresponding author upon reasonable request.
