# Supplementary material for: Patisiran in ATTRv amyloidosis with polyneuropathy: “PatisiranItaly” multicenter observational study
Source: J Neurol. 2025 Feb 15;272(3):209. doi: 10.1007/s00415-025-12950-3 (PMC11829936; doi:10.1007/s00415-025-12950-3)
Supplement: Supplementary file 1 — Supplementary file1 (DOCX 51 KB) [file 415_2025_12950_MOESM1_ESM.docx]

**Patisiran in ATTRv amyloidosis with polyneuropathy: “PatisiranItaly” multicenter observational study**

Vincenzo Di Stefano^1^, Pietro Guaraldi^2^, Angela Romano^3^, Giovanni Antonini^4^, Alessandro Barilaro^5^, Chiara Briani^6^, Marco Burattini^7^, Ilaria Cani^2*^, Giulia Carlini^8^, Marco Ceccanti^9^, Vittoria Cianci^10^, Pietro Cortelli^2*^, Marco Currò Dossi^11^, Daniela Di Lisi^12^, Antonio Di Muzio^13^, Yuri Falzone^14^, Massimiliano Filosto^15,16^, Sabrina Gasverde^17^, Chiara Gemelli^18^, Luca Gentile^19^, Mariangela Goglia^20^, Luca Leonardi^21^, Simone Longhi^22^, Antonio Lotti^5^, Fiore Manganelli^23^, Anna Mazzeo^19^, Giammarco Milella^24^, Giuseppina Novo^12^, Silvia Fenu^25^, Cristina Petrelli^26^, Loris Poli^27^, Luca Guglielmo Pradotto^28,29^, Massimo Russo^19^, Alessandro Salvalaggio^6^, Maria Ausilia Sciarrone^30^, Luigi Selliti^29^, Matteo Tagliapietra^31^, Stefano Tozza^23^, Mara Turri^32^, Lorenzo Verriello^33^, Francesca Vitali^30^, Filippo Brighina^1*^, Marco Luigetti^3,30*^.

^1^ Department of Biomedicine, Neuroscience and Advanced Diagnostics (BIND), University of Palermo, Palermo, Italy.

^2^ IRCCS Istituto delle Scienze Neurologiche di Bologna, Bologna, Italy.

^3^ UOC Neurologia, Fondazione Policlinico Universitario Agostino Gemelli IRCCS, Rome, Italy.

^4^ Department of Neurology Mental Health and Sensory Organs (NESMOS), Faculty of Medicine and Psychology, 'Sapienza' University of Rome and UniCamillus-Saint Camillus International University of Health Sciences, Rome, Italy.

^5^ AOU Careggi and Department of Neurosciences, Drug and Child Health, University of Florence, Florence, Italy.

^6^ Neurology Unit, Department of Neuroscience, University of Padua, Padua, Italy.

^7^ Neurology Unit, Ospedale Santa Croce di Fano, Fano, Italy.

^8^ Neurological Clinic, Department of Experimental and Clinical Medicine, Marche Polytechnic University, Ancona, Italy.

^9^ Department of Human Neuroscience, Sapienza University of Rome, Rome, Italy.

^10^ Neurology Unit, Great Metropolitan Hospital "Bianchi Melacrino Morelli", Reggio Calabria, Italy.

^11^ Department of Neurology, Infermi Hospital, Rimini, Italy.

^12^ Division of Cardiology, University Hospital Paolo Giaccone, Palermo, Italy.

^13^ Department of Neuroscience, Imaging and Clinical Sciences, "G. D'Annunzio" University, Chieti, Italy.

^14^ Division of Neuroscience, Department of Neurology, Institute of Experimental Neurology, San Raffaele Scientific Institute, Milan, Italy.

^15^ Department of Clinical and Experimental Sciences, University of Brescia, Brescia, Italy.

^16^ NeMO-Brescia Clinical Center for Neuromuscular Diseases, Brescia, Italy.

^17^ ASL TO4, Ciriè, Italy.

^18^ IRCCS Ospedale Policlinico San Martino, Genoa, Italy.

^19^ Department of Clinical and Experimental Medicine, University of Messina, Messina, Italy.

^20^ Neuromuscular Diseases Unit, Department of Systems Medicine, Tor Vergata University of Rome, Rome, Italy.

^21^ Neuromuscular and Rare Disease Centre, Neurology Unit, Sant'Andrea Hospital, Rome, Italy.

^22^ Cardiology Unit, Cardiac Thoracic and Vascular Department, IRCCS Azienda Ospedaliero-Universitaria di Bologna, Bologna, Italy.

^23^Department of Neuroscience, Reproductive and Odontostomatological Science, University of Naples 'Federico II', Naples, Italy.

^24^Neurology Unit, Department of Basic Medical Sciences, Neurosciences and Sense Organs, University of Bari Aldo Moro, Bari, Italy.

^25^ S.C. Malattie Neurologiche Rare, Dipartimento di Neuroscienze Cliniche, Fondazione IRCCS Istituto Neurologico Carlo Besta, Milan, Italy.

^26^ Neurology Unit, AV3, ASUR Marche, Macerata, Italy.

^27^ Unit of Neurology, ASST Spedali Civili, 25100 Brescia, Italy.

^28^ Department of Neuroscience "Rita Levi Montalcini", University of Turin, Turin, Italy.

^29^ IRCCS Istituto Auxologico Italiano,  Piancavallo (Vb), Italy.

^30^ Department of Neuroscience, Università Cattolica del Sacro Cuore, Rome, Italy.

^31^ Department of Neuroscience, Biomedicina e Movimento, Università di Verona, Verona, Italy.

^32^ Dipartimento di Neurologia/Stroke Unit, ospedale di Bolzano, Bolzano, Italia.

^33^ Neurology Unit, Department of Neurosciences, University Hospital Santa Maria della Misericordia, Udine, Italy.

* These Authors shared senior authorship.

**Corresponding Author**:

Dr. Marco Luigetti

Dipartimento di Neuroscienze, Organi di Senso e Torace, Fondazione Policlinico Universitario Agostino Gemelli IRCCS

Largo Agostino Gemelli, 8

00168 ROME, ITALY

Tel.: +39-06-30154435 - Fax No.: +39-06-35501909

Email: mluigetti@gmail.com

**Supplementary Figure 1** Patients’ enrollment and the progression of familial amyloid polyneuropathy (FAP) stage throughout the follow-up period with patisiran. Timepoints are as follows: T0 = start of patisiran treatment; T1 = 9 months; T2 = 18 months; T3 = 27 months; T4 = 36 months
