# Supplementary material for: Patisiran in ATTRv amyloidosis with polyneuropathy: “PatisiranItaly” multicenter observational study
Source: J Neurol. 2025 Feb 15;272(3):209. doi: 10.1007/s00415-025-12950-3 (PMC11829936; doi:10.1007/s00415-025-12950-3)
Supplement: Supplementary file 3 — Supplementary file3 (DOCX 26 KB) [file 415_2025_12950_MOESM3_ESM.docx]

**Patisiran in ATTRv amyloidosis with polyneuropathy: “PatisiranItaly” multicenter observational study**

Vincenzo Di Stefano^1^, Pietro Guaraldi^2^, Angela Romano^3^, Giovanni Antonini^4^, Alessandro Barilaro^5^, Chiara Briani^6^, Marco Burattini^7^, Ilaria Cani^2*^, Giulia Carlini^8^, Marco Ceccanti^9^, Vittoria Cianci^10^, Pietro Cortelli^2*^, Marco Currò Dossi^11^, Daniela Di Lisi^12^, Antonio Di Muzio^13^, Yuri Falzone^14^, Massimiliano Filosto^15,16^, Sabrina Gasverde^17^, Chiara Gemelli^18^, Luca Gentile^19^, Mariangela Goglia^20^, Luca Leonardi^21^, Simone Longhi^22^, Antonio Lotti^5^, Fiore Manganelli^23^, Anna Mazzeo^19^, Giammarco Milella^24^, Giuseppina Novo^12^, Silvia Fenu^25^, Cristina Petrelli^26^, Loris Poli^27^, Luca Guglielmo Pradotto^28,29^, Massimo Russo^19^, Alessandro Salvalaggio^6^, Maria Ausilia Sciarrone^30^, Luigi Selliti^29^, Matteo Tagliapietra^31^, Stefano Tozza^23^, Mara Turri^32^, Lorenzo Verriello^33^, Francesca Vitali^30^, Filippo Brighina^1*^, Marco Luigetti^3,30*^.

^1^ Department of Biomedicine, Neuroscience and Advanced Diagnostics (BIND), University of Palermo, Palermo, Italy.

^2^ IRCCS Istituto delle Scienze Neurologiche di Bologna, Bologna, Italy.

^3^ UOC Neurologia, Fondazione Policlinico Universitario Agostino Gemelli IRCCS, Rome, Italy.

^4^ Department of Neurology Mental Health and Sensory Organs (NESMOS), Faculty of Medicine and Psychology, 'Sapienza' University of Rome and UniCamillus-Saint Camillus International University of Health Sciences, Rome, Italy.

^5^ AOU Careggi and Department of Neurosciences, Drug and Child Health, University of Florence, Florence, Italy.

^6^ Neurology Unit, Department of Neuroscience, University of Padua, Padua, Italy.

^7^ Neurology Unit, Ospedale Santa Croce di Fano, Fano, Italy.

^8^ Neurological Clinic, Department of Experimental and Clinical Medicine, Marche Polytechnic University, Ancona, Italy.

^9^ Department of Human Neuroscience, Sapienza University of Rome, Rome, Italy.

^10^ Neurology Unit, Great Metropolitan Hospital "Bianchi Melacrino Morelli", Reggio Calabria, Italy.

^11^ Department of Neurology, Infermi Hospital, Rimini, Italy.

^12^ Division of Cardiology, University Hospital Paolo Giaccone, Palermo, Italy.

^13^ Department of Neuroscience, Imaging and Clinical Sciences, "G. D'Annunzio" University, Chieti, Italy.

^14^ Division of Neuroscience, Department of Neurology, Institute of Experimental Neurology, San Raffaele Scientific Institute, Milan, Italy.

^15^ Department of Clinical and Experimental Sciences, University of Brescia, Brescia, Italy.

^16^ NeMO-Brescia Clinical Center for Neuromuscular Diseases, Brescia, Italy.

^17^ ASL TO4, Ciriè, Italy.

^18^ IRCCS Ospedale Policlinico San Martino, Genoa, Italy.

^19^ Department of Clinical and Experimental Medicine, University of Messina, Messina, Italy.

^20^ Neuromuscular Diseases Unit, Department of Systems Medicine, Tor Vergata University of Rome, Rome, Italy.

^21^ Neuromuscular and Rare Disease Centre, Neurology Unit, Sant'Andrea Hospital, Rome, Italy.

^22^ Cardiology Unit, Cardiac Thoracic and Vascular Department, IRCCS Azienda Ospedaliero-Universitaria di Bologna, Bologna, Italy.

^23^Department of Neuroscience, Reproductive and Odontostomatological Science, University of Naples 'Federico II', Naples, Italy.

^24^Neurology Unit, Department of Basic Medical Sciences, Neurosciences and Sense Organs, University of Bari Aldo Moro, Bari, Italy.

^25^ S.C. Malattie Neurologiche Rare, Dipartimento di Neuroscienze Cliniche, Fondazione IRCCS Istituto Neurologico Carlo Besta, Milan, Italy.

^26^ Neurology Unit, AV3, ASUR Marche, Macerata, Italy.

^27^ Unit of Neurology, ASST Spedali Civili, 25100 Brescia, Italy.

^28^ Department of Neuroscience "Rita Levi Montalcini", University of Turin, Turin, Italy.

^29^ IRCCS Istituto Auxologico Italiano,  Piancavallo (Vb), Italy.

^30^ Department of Neuroscience, Università Cattolica del Sacro Cuore, Rome, Italy.

^31^ Department of Neuroscience, Biomedicina e Movimento, Università di Verona, Verona, Italy.

^32^ Dipartimento di Neurologia/Stroke Unit, ospedale di Bolzano, Bolzano, Italia.

^33^ Neurology Unit, Department of Neurosciences, University Hospital Santa Maria della Misericordia, Udine, Italy.

* These Authors shared senior authorship.

**Corresponding Author**:

Dr. Marco Luigetti

Dipartimento di Neuroscienze, Organi di Senso e Torace, Fondazione Policlinico Universitario Agostino Gemelli IRCCS

Largo Agostino Gemelli, 8

00168 ROME, ITALY

Tel.: +39-06-30154435 - Fax No.: +39-06-35501909

Email: mluigetti@gmail.com

**Supplementary Table 2. Stratification based on disease severity at the start of patisiran.**

|  | **FAP stage at baseline** | |  |
| --- | --- | --- | --- |
|  | ***FAP1***  n = 130 | ***FAP2***  n = 51 | **Comparisons**  **FAP1 *vs* FAP2^a^**  ***p*** |
| **NIS** |  |  |  |
| *T0* | M = 27.0 ± 21.1;  Mdn = 22.0 [IQR 12.0-39.3] | M = 72.4 ± 30.4;  Mdn = 69.0 [IQR 48.0-95.0] | **< 0.001** |
| *T1* | M = 28.9 ± 21.2;  Mdn = 22.0 [IQR 13.0-43.3] | M = 73.3 ± 30.7;  Mdn = 70.5 [IQR 50.0-90.0] | **< 0.001** |
| *T2* | M = 28.5 ± 24.4;  Mdn = 20.0 [IQR 12.0-46.0] | M = 75.5 ± 32.0;  Mdn = 72.0 [IQR 49.5-93.0] | **< 0.001** |
| *T3* | M = 31.8 ± 24.9;  Mdn = 24.5 [IQR 12.5-46.3] | M = 76.7 ± 32.3;  Mdn = 70.8 [IQR 61.3-91.3] | **< 0.001** |
| *T4* | M = 42.2 ± 26.8;  Mdn = 42.0 [IQR 17.0-63.0] | M = 85.8 ± 11.7;  Mdn = 90.0 [IQR 90.0-92.0] | NE |
| **Norfolk QoL-DN** |  |  |  |
| *T0* | M = 34.2 ± 22.4;  Mdn = 33.0 [IQR 15.5-49.5] | M = 70.2 ± 18.0;  Mdn = 72.5 [IQR 61.0-82.0] | **< 0.001** |
| *T1* | M = 34.0 ± 23.0;  Mdn = 30.5 [IQR 15.5-48.0] | M = 66.3 ± 22.4;  Mdn = 66.0 [IQR 50.0-79.0] | **< 0.001** |
| *T2* | M = 34.1 ± 26.6;  Mdn = 26.0 [IQR 15.0-50.5] | M = 65.8 ± 20.7;  Mdn = 69.0 [IQR 58.0-81.0] | **< 0.001** |
| *T3* | M = 34.0 ± 23.5;  Mdn = 29.0 [IQR 17.0-51.0] | M = 66.6 ± 20.0;  Mdn = 74.0 [IQR 48.0-82.0] | **< 0.001** |
| *T4* | M = 32.9 ± 23.1;  Mdn = 26.5 [IQR 16.5-45.0] | M = 88.4 ± 12.4;  Mdn = 88.0 [IQR 80.0-94.0] | NE |
| **CADT in M** |  |  |  |
| *T0* | M = 16.7 ± 3.3;  Mdn = 18.0 [IQR 16.0-20.0] | M = 14.5 ± 4.1;  Mdn = 15.5 [IQR 11.0-18.0] | **0.022** |
| *T1* | M = 16.7 ± 2.9;  Mdn = 17.0 [IQR 15.0-20.0] | M = 15.0 ± 3.5;  Mdn = 15.5 [IQR 12.0-18.0] | 0.065 |
| *T2* | M = 17.1 ± 2.4;  Mdn = 16.5 [IQR 16.0-20.0] | M = 14.6 ± 3.0;  Mdn = 14.0 [IQR 12.0-16.0] | **0.010** |
| *T3* | M = 16.9 ± 2.9;  Mdn = 16.0 [IQR 15.0-20.0] | M = 15.8 ± 2.3;  Mdn = 16.0 [IQR 14.0-17.0] | 0.277 |
| *T4* | M = 15.8 ± 1.7;  Mdn = 15.5 [IQR 14.5-17.0] | M = 16.0 ± 2.9;  Mdn = 16.5 [IQR 14.0-18.0] | NE |
| **CADT in F** |  |  |  |
| *T0* | M = 14.6 ± 1.8;  Mdn = 15.0 [IQR 14.0-16.0] | M = 12.1 ± 3.6;  Mdn = 12.0 [IQR 9.0-16.0] | NE |
| *T1* | M = 14.0 ± 2.3;  Mdn = 14.0 [IQR 13.0-16.0] | M = 13.0 ± 2.5;  Mdn = 12.5 [IQR 11.0-16.0] | NE |
| *T2* | M = 14.0 ± 1.9;  Mdn = 14.0 [IQR 13.0-16.0] | M = 13.7 ± 2.1;  Mdn = 13.0 [IQR 12.0-16.0] | NE |
| *T3* | M = 14.1 ± 1.7;  Mdn = 14.0 [IQR 13.0-16.0] | M = 14.0 ± 2.8;  Mdn = 14.0 [IQR 12.0-16.0] | NE |
| *T4* | M = 14.3 ± 1.5;  Mdn = 14.0 [IQR 13.0-16.0] | M = 12.0 ± 0.0;  Mdn = 12.0 [IQR 12.0-12.0] | NE |
| **IVS** (*mm*) |  |  |  |
| *T0* | M = 14.5 ± 4.2;  Mdn = 14.4 [IQR 11.0-17.1] | M = 14.6 ± 3.5;  Mdn = 14.0 [IQR 12.4-16.5] | 0.825 |
| *T1* | M = 14.3 ± 3.9;  Mdn = 14.0 [IQR 12.0-17.2] | M = 14.7 ± 4.0;  Mdn = 15.0 [IQR 13.0-17.0] | 0.613 |
| *T2* | M = 14.3 ± 3.5;  Mdn = 14.0 [IQR 12.0-17.0] | M = 15.4 ± 3.1;  Mdn = 15.0 [IQR 13.0-18.0] | 0.237 |
| *T3* | M = 14.0 ± 3.1;  Mdn = 13.0 [IQR 12.0-16.0] | M = 15.2 ± 3.1;  Mdn = 16.0 [IQR 13.0-17.0] | 0.243 |
| *T4* | M = 14.4 ± 2.8;  Mdn = 13.0 [IQR 13.0-15.0] | M = 14.4 ± 3.2;  Mdn = 14.0 [IQR 13.0-17.0] | NE |
| *T0* | M = 999.3 ± 1555.3;  Mdn = 365.5 [IQR 101.0-1105.0] | M = 1843.0 ± 2500.1;  Mdn = 1006.5 [IQR 357.0-2045.0] | **0.003** |
| *T1* | M = 1461.2 ± 3528.2;  Mdn = 245.0 [IQR 84.0-1169.0] | M = 3305.9 ± 7617.2;  Mdn = 673.0 [IQR 291.0-1800.0] | **0.009** |
| *T2* | M = 2124.8 ± 5658.1;  Mdn = 342.5 [IQR 77.0-1568.0] | M = 1402.8 ± 2578.7;  Mdn = 598.5 [IQR 252.0-1488.0] | 0.156 |
| *T3* | M = 1256.3 ± 2927.3;  Mdn = 235.0 [IQR 78.0-1123.0] | M = 996.7 ± 1108.4;  Mdn = 420.0 [IQR 338.0-1177.0] | 0.119 |
| *T4* | M = 549.5 ± 562.6;  Mdn = 345.5 [IQR 90.0-950.0] | M = 1417.3 ± 1813.5;  Mdn = 330.5 [IQR 231.0-3280.0] | NE |

**Supplementary table 2.** Main clinical features of the study cohort at each time point (from T0 to T4), stratified on the basis of the FAP stage at baseline (i.e., the start of patisiran treatment). *n* refers to the count in each group at the baseline evaluation (T0).

Variables are reported as mean (M) ± standard deviation; median (Mdn) and interquartile range (IQR), rounded to the first decimal place.

NIS, Neuropathy Impairment Score; Norfolk QoL-DN, Norfolk Quality of Life‐Diabetic Neuropathy questionnaire; CADT, Compound Autonomic Dysfunction Test; M, males; F, females; IVS, interventricular septum; NT-proBNP, N-terminal pro B-type natriuretic peptide. NE: not evaluable.

^a^ Comparisons between FAP1 and FAP2 patients were evaluated by the Mann-Whitney U test. Significant *p* values are displayed in bold.
