# Supplementary material for: Patisiran in ATTRv amyloidosis with polyneuropathy: “PatisiranItaly” multicenter observational study
Source: J Neurol. 2025 Feb 15;272(3):209. doi: 10.1007/s00415-025-12950-3 (PMC11829936; doi:10.1007/s00415-025-12950-3)
Supplement: Supplementary file 4 — Supplementary file4 (DOCX 29 KB) [file 415_2025_12950_MOESM4_ESM.docx]

**Patisiran in ATTRv amyloidosis with polyneuropathy: “PatisiranItaly” multicenter observational study**

Vincenzo Di Stefano^1^, Pietro Guaraldi^2^, Angela Romano^3^, Giovanni Antonini^4^, Alessandro Barilaro^5^, Chiara Briani^6^, Marco Burattini^7^, Ilaria Cani^2*^, Giulia Carlini^8^, Marco Ceccanti^9^, Vittoria Cianci^10^, Pietro Cortelli^2*^, Marco Currò Dossi^11^, Daniela Di Lisi^12^, Antonio Di Muzio^13^, Yuri Falzone^14^, Massimiliano Filosto^15,16^, Sabrina Gasverde^17^, Chiara Gemelli^18^, Luca Gentile^19^, Mariangela Goglia^20^, Luca Leonardi^21^, Simone Longhi^22^, Antonio Lotti^5^, Fiore Manganelli^23^, Anna Mazzeo^19^, Giammarco Milella^24^, Giuseppina Novo^12^, Silvia Fenu^25^, Cristina Petrelli^26^, Loris Poli^27^, Luca Guglielmo Pradotto^28,29^, Massimo Russo^19^, Alessandro Salvalaggio^6^, Maria Ausilia Sciarrone^30^, Luigi Selliti^29^, Matteo Tagliapietra^31^, Stefano Tozza^23^, Mara Turri^32^, Lorenzo Verriello^33^, Francesca Vitali^30^, Filippo Brighina^1*^, Marco Luigetti^3,30*^.

^1^ Department of Biomedicine, Neuroscience and Advanced Diagnostics (BIND), University of Palermo, Palermo, Italy.

^2^ IRCCS Istituto delle Scienze Neurologiche di Bologna, Bologna, Italy.

^3^ UOC Neurologia, Fondazione Policlinico Universitario Agostino Gemelli IRCCS, Rome, Italy.

^4^ Department of Neurology Mental Health and Sensory Organs (NESMOS), Faculty of Medicine and Psychology, 'Sapienza' University of Rome and UniCamillus-Saint Camillus International University of Health Sciences, Rome, Italy.

^5^ AOU Careggi and Department of Neurosciences, Drug and Child Health, University of Florence, Florence, Italy.

^6^ Neurology Unit, Department of Neuroscience, University of Padua, Padua, Italy.

^7^ Neurology Unit, Ospedale Santa Croce di Fano, Fano, Italy.

^8^ Neurological Clinic, Department of Experimental and Clinical Medicine, Marche Polytechnic University, Ancona, Italy.

^9^ Department of Human Neuroscience, Sapienza University of Rome, Rome, Italy.

^10^ Neurology Unit, Great Metropolitan Hospital "Bianchi Melacrino Morelli", Reggio Calabria, Italy.

^11^ Department of Neurology, Infermi Hospital, Rimini, Italy.

^12^ Division of Cardiology, University Hospital Paolo Giaccone, Palermo, Italy.

^13^ Department of Neuroscience, Imaging and Clinical Sciences, "G. D'Annunzio" University, Chieti, Italy.

^14^ Division of Neuroscience, Department of Neurology, Institute of Experimental Neurology, San Raffaele Scientific Institute, Milan, Italy.

^15^ Department of Clinical and Experimental Sciences, University of Brescia, Brescia, Italy.

^16^ NeMO-Brescia Clinical Center for Neuromuscular Diseases, Brescia, Italy.

^17^ ASL TO4, Ciriè, Italy.

^18^ IRCCS Ospedale Policlinico San Martino, Genoa, Italy.

^19^ Department of Clinical and Experimental Medicine, University of Messina, Messina, Italy.

^20^ Neuromuscular Diseases Unit, Department of Systems Medicine, Tor Vergata University of Rome, Rome, Italy.

^21^ Neuromuscular and Rare Disease Centre, Neurology Unit, Sant'Andrea Hospital, Rome, Italy.

^22^ Cardiology Unit, Cardiac Thoracic and Vascular Department, IRCCS Azienda Ospedaliero-Universitaria di Bologna, Bologna, Italy.

^23^Department of Neuroscience, Reproductive and Odontostomatological Science, University of Naples 'Federico II', Naples, Italy.

^24^Neurology Unit, Department of Basic Medical Sciences, Neurosciences and Sense Organs, University of Bari Aldo Moro, Bari, Italy.

^25^ S.C. Malattie Neurologiche Rare, Dipartimento di Neuroscienze Cliniche, Fondazione IRCCS Istituto Neurologico Carlo Besta, Milan, Italy.

^26^ Neurology Unit, AV3, ASUR Marche, Macerata, Italy.

^27^ Unit of Neurology, ASST Spedali Civili, 25100 Brescia, Italy.

^28^ Department of Neuroscience "Rita Levi Montalcini", University of Turin, Turin, Italy.

^29^ IRCCS Istituto Auxologico Italiano,  Piancavallo (Vb), Italy.

^30^ Department of Neuroscience, Università Cattolica del Sacro Cuore, Rome, Italy.

^31^ Department of Neuroscience, Biomedicina e Movimento, Università di Verona, Verona, Italy.

^32^ Dipartimento di Neurologia/Stroke Unit, ospedale di Bolzano, Bolzano, Italia.

^33^ Neurology Unit, Department of Neurosciences, University Hospital Santa Maria della Misericordia, Udine, Italy.

* These Authors shared senior authorship.

**Corresponding Author**:

Dr. Marco Luigetti

Dipartimento di Neuroscienze, Organi di Senso e Torace, Fondazione Policlinico Universitario Agostino Gemelli IRCCS

Largo Agostino Gemelli, 8

00168 ROME, ITALY

Tel.: +39-06-30154435 - Fax No.: +39-06-35501909

Email: mluigetti@gmail.com

**Supplementary Table 3. Stratification based on genotype**

|  | **p.Phe84Leu**  n = 54 | **p.Ile88Leu + p.Val142Ile**  n = 45 | **p.Val50Met**  n = 37 | **p.Glu109Gln**  n = 15 | **Comparisons between groups**^a^ | |
| --- | --- | --- | --- | --- | --- | --- |
|  |  |  |  |  | ***p*** | **Pairwise comparisons**^b^  ***adj. p*** |
| **NIS** |  |  |  |  |  |  |
| *T0* | M = 54.4 ± 33.5;  Mdn = 48.0 [IQR 34.0-73.5] | M = 20.9 ± 15.6;  Mdn = 15.0 [IQR 10.0-29.0] | M = 49.4 ± 31.5;  Mdn = 46.0 [IQR 22.0-68.0] | M = 39.5 ± 27.0;  Mdn = 28.0 [IQR 20.0-48.0] | **< 0.001** | I88L+V122I *vs* F64L < 0.001  I88L+V122I *vs* V30M < 0.001 |
| *T1* | M = 53.7 ± 31.3;  Mdn = 49.0 [IQR 32.3-76.0] | M = 21.0 ± 16.3;  Mdn = 16.0 [IQR 11.0-25.0] | M = 52.7 ± 33.3;  Mdn = 52.0 [IQR 24.0-70.0] | M = 36.4 ± 24.8;  Mdn = 27.0 [IQR 20.0-46.0] | **< 0.001** | I88L+V122I *vs* F64L < 0.001  I88L+V122I *vs* V30M < 0.001 |
| *T2* | M = 50.3 ± 33.6;  Mdn = 48.0 [IQR 22.0-70.5] | M = 18.5 ± 11.7;  Mdn = 14.5 [IQR 12.0-20.0] | M = 60.6 ± 34.4;  Mdn = 60.3 [IQR 42.0-90.0] | M = 36.8 ± 31.6;  Mdn = 22.0 [IQR 15.0-62.0] | **< 0.001** | I88L+V122I *vs* F64L = 0.001  I88L+V122I *vs* V30M < 0.001 |
| *T3* | M = 56.8 ± 31.2;  Mdn = 58.0 [IQR 40.0-74.0] | M = 21.0 ± 20.6;  Mdn = 12.0 [IQR 10.0-21.0] | M = 57.1 ± 34.6;  Mdn = 62.5 [IQR 28.0-65.0] | M = 42.9 ± 33.2;  Mdn = 29.0 [IQR 17.0-88.0] | **0.010** | I88L+V122I *vs* F64L = 0.012  I88L+V122I *vs* V30M = 0.025 |
| *T4* | M = 67.0 ± 23.1;  Mdn = 69.0 [IQR 48.0-86.0] | *No observations* | M = 55.4 ± 29.2;  Mdn = 64.0 [IQR 42.0-65.0] | M = 66.3 ± 42.7;  Mdn = 90.0 [IQR 17.0-92.0] | NE |  |
| **Norfolk QoL-DN** | |  |  |  |  |  |
| *T0* | M = 55.5 ± 25.7;  Mdn = 56.0 [IQR 41.0-74.0] | M = 31.6 ± 24.3;  Mdn = 26.0 [IQR 12.0-46.0] | M = 48.7 ± 24.9;  Mdn = 50.0 [IQR 31.0-67.0] | M = 46.2 ± 28.1;  Mdn = 46.0 [IQR 16.0-73.0] | **< 0.001** | I88L+V122I *vs* F64L < 0.001  I88L+V122I *vs* V30M = 0.028 |
| *T1* | M = 54.2 ± 28.1;  Mdn = 54.0 [IQR 38.0-72.0] | M = 28.9 ± 21.8;  Mdn = 25.0 [IQR 11.0-45.0] | M = 47.8 ± 27.3;  Mdn = 45.0 [IQR 25.0-67.0] | M = 39.4 ± 22.3;  Mdn = 35.0 [IQR 20.0-60.0] | **< 0.001** | I88L+V122I *vs* F64L < 0.001  I88L+V122I *vs* V30M = 0.020 |
| *T2* | M = 49.9 ± 26.9;  Mdn = 52.5 [IQR 25.0-70.0] | M = 26.7 ± 25.3;  Mdn = 24.0 [IQR 12.0-35.0] | M = 49.9 ± 31.6;  Mdn = 48.5 [IQR 23.5-80.0] | M = 36.8 ± 23.9;  Mdn = 30.5 [IQR 17.5-60.5] | **0.010** | I88L+V122I *vs* F64L = 0.010  I88L+V122I *vs* V30M = 0.046 |
| *T3* | M = 53.5 ± 27.4;  Mdn = 58.0 [IQR 44.0-72.0] | M = 22.5 ± 14.8;  Mdn = 18.0 [IQR 9.0-34.0] | M = 52.8 ± 27.5;  Mdn = 38.0 [IQR 29.0-82.0] | M = 37.6 ± 27.4;  Mdn = 23.0 [IQR 19.0-74.0] | **0.016** | I88L+V122I *vs* F64L = 0.030  I88L+V122I *vs* V30M = 0.036 |
| *T4* | M = 55.3 ± 28.8;  Mdn = 64.0 [IQR 34.5-76.0] | *No observations* | M = 60.0 ± 40.2;  Mdn = 58.0 [IQR 27.0-93.0] | M = 63.7 ± 47.4;  Mdn = 88.0 [IQR 9.0-94.0] | NE |  |
| **CADT in M** | |  |  |  |  |  |
| *T0* | M = 15.0 ± 3.3;  Mdn = 16.0 [IQR 12.0-18.0] | M = 18.3 ± 2.4;  Mdn = 19.5 [IQR 17.0-20.0] | M = 15.3 ± 4.1;  Mdn = 16.5 [IQR 12.0-18.0] | M = 15.0 ± 4.7;  Mdn = 16.0 [IQR 13.0-18.0] | **0.004** | I88L+V122I *vs* F64L = 0.002 |
| *T1* | M = 15.6 ± 2.7;  Mdn = 16.0 [IQR 14.0-18.0] | M = 17.8 ± 3.1;  Mdn = 20.0 [IQR 16.0-20.0] | M = 14.4 ± 3.3;  Mdn = 14.0 [IQR 12.0-16.0] | M = 16.8 ± 2.3;  Mdn = 16.0 [IQR 16.0-18.0] | **0.006** | I88L+V122I *vs* F64L = 0.036  I88L+V122I *vs* V30M = 0.007 |
| *T2* | M = 15.6 ± 2.4;  Mdn = 16.0 [IQR 14.0-16.0] | M = 18.6 ± 1.7;  Mdn = 19.0 [IQR 18.0-20.0] | M = 15.1 ± 3.0;  Mdn = 16.0 [IQR 12.0-17.0] | M = 17.0 ± 4.2;  Mdn = 17.0 [IQR 14.0-20.0] | **0.025** | I88L+V122I *vs* F64L = 0.038  I88L+V122I *vs* V30M = 0.042 |
| *T3* | M = 15.6 ± 1.4;  Mdn = 16.0 [IQR 15.0-16.0] | M = 17.6 ± 2.6;  Mdn = 18.0 [IQR 16.0-20.0] | M = 15.4 ± 3.4;  Mdn = 16.0 [IQR 12.0-19.0] | M = 17.0 ± 4.2;  Mdn = 17.0 [IQR 14.0-20.0] | NE |  |
| *T4* | M = 15.5 ± 1.3;  Mdn = 15.5 [IQR 14.5-16.5] | *No observations* | M = 15.7 ± 3.5;  Mdn = 16.0 [IQR 12.0-19.0] | *No observations* | NE |  |
| **CADT in F** | |  |  |  |  |  |
| *T0* | M = 15.0 ± 0.8;  Mdn = 15.0 [IQR 14.5-15.5] | M = 16.0 ± 0.0;  Mdn = 16.0 [IQR 16.0-16.0] | M = 13.8 ± 2.9;  Mdn = 15.0 [IQR 12.0-16.0] | M = 13.3 ± 3.3;  Mdn = 14.0 [IQR 12.0-16.0] | NE |  |
| *T1* | M = 14.3 ± 0.6;  Mdn = 14.0 [IQR 14.0-15.0] | M = 15.0 ± 1.5;  Mdn = 16.0 [IQR 13.0-16.0] | M = 13.6 ± 2.1;  Mdn = 13.0 [IQR 12.0-16.0] | M = 14.2 ± 2.4;  Mdn = 15.0 [IQR 13.0-16.0] | NE |  |
| *T2* | M = 12.5 ± 3.5;  Mdn = 12.5 [IQR 10.0-15.0] | M = 15.0 ± 2.0;  Mdn = 16.0 [IQR 14.0-16.0] | M = 13.0 ± 0.8;  Mdn = 13.0 [IQR 12.5-13.5] | M = 13.7 ± 2.1;  Mdn = 13.0 [IQR 12.0-16.0] | NE |  |
| *T3* | M = 16.0 ± .;  Mdn = 16.0 [IQR 16.0-16.0] | M = 16.0 ± 0.0;  Mdn = 16.0 [IQR 16.0-16.0] | M = 13.8 ± 1.3;  Mdn = 14.0 [IQR 13.0-14.5] | M = 14.0 ± 2.8;  Mdn = 14.0 [IQR 12.0-16.0] | NE |  |
| *T4* | *No observations* | *No observations* | M = 15.0 ± 1.4;  Mdn = 15.0 [IQR 14.0-16.0] | M = 12.0 ± 0.0;  Mdn = 12.0 [IQR 12.0-12.0] | NE |  |
| **IVS** (*mm*) | |  |  |  |  |  |
| *T0* | M = 12.2 ± 2.1;  Mdn = 12.0 [IQR 11.0-13.0] | M = 17.2 ± 3.6;  Mdn = 17.0 [IQR 15.0-19.0] | M = 14.6 ± 3.7;  Mdn = 14.5 [IQR 12.0-18.0] | M = 14.2 ± 3.9;  Mdn = 14.0 [IQR 13.0-15.0] | **< 0.001** | I88L+V122I *vs* F64L < 0.001  F64L *vs* V30M = 0.050 |
| *T1* | M = 12.4 ± 2.3;  Mdn = 12.0 [IQR 11.0-13.0] | M = 16.5 ± 3.4;  Mdn = 17.0 [IQR 16.0-18.4] | M = 14.6 ± 3.6;  Mdn = 14.5 [IQR 12.0-18.0] | M = 13.8 ± 4.4;  Mdn = 15.0 [IQR 12.0-16.0] | **< 0.001** | I88L+V122I *vs* F64L < 0.001 |
| *T2* | M = 12.6 ± 2.1;  Mdn = 12.0 [IQR 11.0-13.5] | M = 17.4 ± 1.9;  Mdn = 17.0 [IQR 16.0-18.0] | M = 15.4 ± 4.3;  Mdn = 16.0 [IQR 13.0-19.0] | M = 16.2 ± 2.6;  Mdn = 16.0 [IQR 15.0-17.0] | **< 0.001** | I88L+V122I *vs* F64L < 0.001 |
| *T3* | M = 12.2 ± 1.2;  Mdn = 12.0 [IQR 12.0-12.0] | M = 16.8 ± 2.5;  Mdn = 17.0 [IQR 16.0-19.0] | M = 15.0 ± 4.0;  Mdn = 14.5 [IQR 12.0-19.0] | M = 17.2 ± 1.6;  Mdn = 17.0 [IQR 16.0-17.0] | NE |  |
| *T4* | M = 13.5 ± 1.3;  Mdn = 13.5 [IQR 12.5-14.5] | *No observations* | M = 14.0 ± 4.6;  Mdn = 13.0 [IQR 10.0-19.0] | M = 17.5 ± 0.7;  Mdn = 17.5 [IQR 17.0-18.0] | NE |  |
| **NT-proBNP** (ng/L) | |  |  |  |  |  |
| *T0* | M = 650.6 ± 1239.8;  Mdn = 163.5 [IQR 64.0-878.0] | M = 2391.3 ± 2237.4;  Mdn = 1831.0  [IQR 529.0-3378.0] | M = 802.2 ± 791.7;  Mdn = 600.0  [IQR 240.0-1093.0] | M = 1016.6 ± 1372.9;  Mdn = 623.0  [IQR 116.0-1428.0] | **< 0.001** | I88L+V122I *vs* F64L < 0.001 |
| *T1* | M = 1655.3 ± 5440.2;  Mdn = 162.0 [IQR 60.0-544.0] | M = 4285.6 ± 5590.0;  Mdn = 2371.0  [IQR 470.0-8270.0] | M = 799.3 ± 886.2;  Mdn = 518.0  [IQR 160.0-1041.0] | M = 671.3 ± 596.1;  Mdn = 371.0  [IQR 137.0-1169.0] | **< 0.001** | I88L+V122I *vs* F64L < 0.001 |
| *T2* | M = 363.1 ± 438.9;  Mdn = 109.3 [IQR 67.0-592.0] | M = 7404.1 ± 10136.5;  Mdn = 3441.0  [IQR 605.0-12957.0] | M = 718.2 ± 798.3;  Mdn = 531.7  [IQR 232.0-890.0] | M = 1076.3 ± 770.7;  Mdn = 1200.0  [IQR 210.0-1672.0] | **0.003** | I88L+V122I *vs* F64L = 0.002 |
| *T3* | M = 445.4 ± 530.2;  Mdn = 168.0 [IQR 78.0-467.0] | M = 4217.3 ± 5766.1;  Mdn = 2012.5  [IQR 620.0-5112.0] | M = 789.7 ± 1032.8;  Mdn = 410.0  [IQR 235.0-955.0] | M = 1409.4 ± 1190.0;  Mdn = 1123.0  [IQR 231.0-2264.0] | 0.057 | N/A |
| *T4* | M = 545.0 ± 649.5;  Mdn = 307.0  [IQR 117.5-972.5] | *No observations* | M = 460.5 ± 335.0;  Mdn = 330.5  [IQR 244.5-676.5] | M = 2563.0 ± 2066.0;  Mdn = 3280.0  [IQR 234.0-4175.0] | NE |  |

**Supplementary table 3.** Main clinical features of the study cohort at each time point (from T0 to T4), stratified based on *TTR* variant. *n* refers to the count in each group at the baseline evaluation (T0).

Variables are reported as mean (M) ± standard deviation; median (Mdn) and interquartile range (IQR), rounded to the first decimal place.

NIS, Neuropathy Impairment Score; Norfolk QoL-DN, Norfolk Quality of Life‐Diabetic Neuropathy questionnaire; CADT, Compound Autonomic Dysfunction Test; M, males; F, females; IVS, interventricular septum; NT-proBNP, N-terminal pro B-type natriuretic peptide. NE: not evaluable. N/A: not applicable.

^a^ Comparisons between groups were evaluated by the Kruskal-Wallis H test.

^b^ Post-hoc pairwise comparisons, where pertinent, were assessed using the Dunn's test with Bonferroni correction for multiple tests. Only statistically significant post-hoc tests are displayed. Adjusted p-values are reported.
